# Role of somatic cells on dairy processes and products: a review

**DOI:** 10.1007/s13594-014-0176-3

**Published:** 2014-07-17

**Authors:** N. Li, R. Richoux, M. Boutinaud, P. Martin, V. Gagnaire

**Affiliations:** 1 INRA, UMR 1253, Science et Technologie du Lait et de l’Œuf, 65 rue de Saint Brieuc, F-35042 Rennes, France; 2 Agrocampus Ouest, UMR 1253, Science et Technologie du Lait et de l’Œuf, 65 rue de Saint Brieuc, 35042 Rennes, France; 3 Actalia, BP 50915, 35009 Rennes, Cedex France; 4 INRA, UMR 1348, Physiologie, Environnement et Génétique pour l’Animal et les Systèmes d’Élevage, 35590 Saint Gilles, France; 5 Agrocampus Ouest, UMR 1348, Physiologie, Environnement et Génétique pour l’Animal et les Systèmes d’Élevage, 35590 Saint Gilles, France; 6 INRA, UMR 1313, Génétique Animale et Biologie Intégrative, 78350 Jouy-en-Josas, France; 7 AgroParisTech, UMR 1313, Génétique Animale et Biologie Intégrative, 78350 Jouy-en-Josas, France

**Keywords:** Milk, Somatic cells, Enzymes, Cathepsin D, Cheese

## Abstract

Somatic cells are an important component naturally present in milk, and somatic cell count is used as an indicator of udder health and milk quality. The role of somatic cells in dairy processes and products is ill-defined in most studies because the role of these cells combines also the concomitance of physicochemical modifications of milk, bacterial count, and the udder inflammation in the presence of high somatic cell count. The aim of this review is to focus on the role of somatic cells themselves and of endogenous enzymes from somatic cells in milk, in dairy transformation processes, and in characteristics of final products overcoming biases due to other factors. The immune function of somatic cells in the udder defense and their protective role in milk will be primarily considered. Different characteristics of milk induced by various somatic cell counts, types, and their endogenous enzymes influencing directly the technological properties of milk and the final quality of dairy products will be discussed as well. By comparing methods used in other studies and eliminating biases due to other factors not considered in these studies, a new approach has been suggested to evaluate the effective role of somatic cells on dairy processes and products. In addition, this new approach allows the characterization of somatic cells and their endogenous enzymes and, in future research, will allow the clarification of mechanisms involved in the release of these components from somatic cells during dairy processes, particularly in cheese technologies.

## Introduction

Milk is known to be a high-value nutritional biological fluid composed of water, proteins, fat, sugars, minerals, etc. Other important components existing naturally in raw milk are somatic cells (SCs), and the predominant cell type, besides shed epithelial cells, in most species is leucocytes, including macrophages, polymorphonuclear neutrophils cells (PMNs), and lymphocytes (Boutinaud and Jammes 2002).

The amount of SCs, usually called somatic cell count (SCC), in milk is used as an important indicator of udder health since SCs are involved in protecting the mammary gland from infection as part of the innate immune system. SCC in milk is influenced by many factors, such as animal species, milk production level, lactation stage, and also the individual and environmental factors as well as management practices (Rupp et al. 2000). Though SCC is subjected to variation, it is still used as an indicator of milk quality in several species, especially in ruminant and human (Hunt et al. 2013; Sharma et al. 2011). Taking cow milk as an example, when SCC >2 × 10^5^ cells.mL^−1^, the udder is considered to be infected, and when SCC >4 × 10^5^ cells.mL^−1^, the milk is deemed unfit for human consumption in the European Union (EU). The legal SCC threshold for milk acceptance in dairy industries varies in different countries, e.g., the values for bovine milk in Germany, Canada, and the USA are 1 × 10^5^, 5 × 10^5^, and 7.5 × 10^5^ cells.mL^−1^, respectively (Olechnowicz and Jaskowski 2012; Schwarz et al. 2011). For caprine and ovine milk, the cutoff value is 1 × 10^6^ in the USA but is not yet defined in the EU (Council Directive 92/46/EEC 1992).

Whether SC is “a friend or a foe” in the dairy field remains a question (Souza et al. 2012). Generally, SCs until now have been considered as negative. High SCC is associated with udder inflammation, which leads to bacteriological problems in milk, an alteration of milk composition, and finally, the major modifications of dairy product characteristics compared to the normal values (Le Maréchal et al. 2011; Lindmark-Mansson et al. 2006; Raynal-Ljutovac et al. 2007; Sharma et al. 2011). However, besides their immune function in the udder and protective functions in milk, SCs have recently been shown to influence, in a positive way, the composition and technological properties of dairy products, thus participating in the final quality of dairy products through their endogenous enzymes (Sanchez-Macias et al. 2013; Souza et al. 2012).

The role of SCs is generally assessed as a global effect, although the influence of the other factors has not been considered separately, and then, includes intrinsic characteristics of milk modified by the inflammation of the mammary gland, consequences on milk biosynthesis and secretion, and bacterial count (Albenzio et al. 2004; O’Farrell et al. 2002; Raynal-Ljutovac et al. 2007). Few research studies until now have given a clear view of the role of SCs without the combination of the other causative factors. This review focuses on the state of our knowledge of SCs in the dairy field and on their effective role in dairy processes and products.

## Somatic cells and udder defense mechanism

### The immune function of somatic cells in the udder

SCs are known to be one of the major defense components of the mammary gland against disease or intramammary infections (Paape et al. 1979, 2002; Sharma et al. 2011). The four main cell types composing SC, namely, macrophages, PMNs, lymphocytes, and epithelial cells, are briefly presented in Table 1.

**Table 1:**
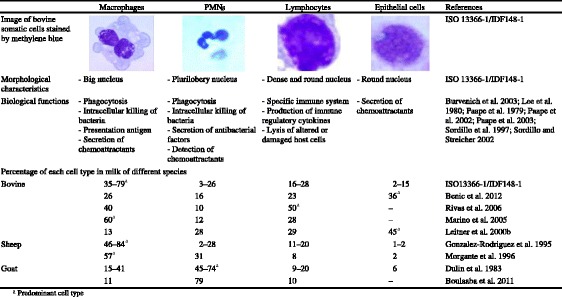
Characteristics and composition of somatic cells in healthy milk of different species

Macrophages are generally the predominant cell type in healthy cow milk. They can fight against bacterial invasion quickly by engulfing action. In the case of infection, macrophages release chemical messengers or chemoattractants that are detected by PMNs and direct PMNs in turn towards the infection site. Both macrophages and PMNs can ingest microbial cells by phagocytosis and have an essential role in the innate immune system. Moreover, macrophages participate in the specific immunity as do lymphocytes (Burvenich et al. 2003).

PMNs can be recruited and increase milk SCC when the infection continues. They can be present to a large extent in mastitic milk, even up to 92 % in bovine milk (Paape et al. 1979). When PMNs arrive at the site of infection, they phagocyte microorganisms and kill them by using a combination of oxidative and non-oxidative mechanisms (Pham 2006).

Lymphocytes have a determinant role in the specific immune system. They are the only cells able to recognize the antigens through specific membrane receptors for invading pathogens (Sordillo et al. 1997).

Mammary epithelial cells are the cells that produce milk. They are shed from the mammary epithelium during lactation (Boutinaud and Jammes 2002). Prior to 1980, they were confused with macrophages because of their similar morphological traits and finally discovered in milk by electron microscopy (Lee et al. 1980). The epithelial cells are the first defense line of the mammary glands, and they may participate in the immunity of neonates in different species (Boutinaud and Jammes 2002). This type of cell is often detected below 15 % (Table 1) as also confirmed in other studies (Ben Chedly et al. 2011; Boutinaud et al. 2008; Rankl 2004). The mammary epithelial feature of these cells has been further determined in cows and goats using transcriptomic and proteomic analyses, demonstrating that their content in mRNA and proteins, such as cytokeratin and enzymes involved in milk synthesis, is specific to mammary epithelial cells (Ben Chedly et al. 2011; Boutinaud et al. 2008; Janjanam et al. 2013).

### The protective function of somatic cells in milk

Besides the immune defense role in the udder, SCs can continue their protective function in milk. Additionally, some components identified as being from SCs are present in milk and also help to enhance the host defense. For example, PMNs have bactericidal and respiratory burst activities and they can eliminate the invading bacteria by releasing reactive oxygen species (ROS) and granular enzymes (Paape et al. 2002). Some antibacterial proteins identified in bovine milk also arise from SC such as macrophage scavenger receptor types I and II, PMN peptidoglycan recognition protein and lymphocyte cytosolic protein 1 and cathelicidins. They can continue to exert their protective properties when they are in skim milk, whey, or milk fat globule membranes (Hettinga et al. 2011; Smolenski et al. 2006). Recently, information was obtained on the protein changes of the bovine and goat innate immune system following exposure to lipopolysaccharide (LPS) from the cell walls of gram-negative *Escherichia coli*, leading to an increase in SCC without having the contribution of bacteria themselves (Hinz et al. 2012; Olumee-Shabon et al. 2013). In both ruminant species, an increase in the antimicrobial proteins was observed and more precisely of cathelicidins, which are involved in the degranulation of PMNs. In caprine milk, acute-phase proteins, haptoglobin, and serum amyloid A were shown in milk collected after 18 h after infusion with LPS.

The enzymes initially from SCs can be released or secreted in milk and are therefore considered as endogenous SC enzymes and play an important protective role in milk. The role of the lysozyme, one SC endogenous enzyme, is well known for the ability to destroy bacteria (Paape et al. 2003). Some proteinases from PMNs, such as cathepsin G, elastase, and proteinase 3, have antimicrobial activities during phagocytosis of invading microorganisms. They could also contribute, after release, to the extracellular killing of microorganisms by cleaving their bacterial virulence factors as shown in mice (Pham 2006). Catalase, an endogenous enzyme from PMNs, is one of the main antioxidant enzymes in milk and is suspected of being responsible for changed redox potential of milk that limited the survival capability of microorganisms (Hamed et al. 2008).

Even if the release mechanism of these protective components from SCs is not fully understood yet, the release of these SC endogenous proteins including enzymes has already been mentioned in the last decade, as described in Section 3.1. The contribution of these SC endogenous enzymes to dairy transformation processing and final characteristics of products has been considered previously by several authors, and this issue will be discussed in Section 4.1.

### Identification of somatic cell types

Several methods have been developed to differentiate SCs in milk, and among them, cytology methods are mainly used to visually identify the main cell types by optical microscopy (Baumert et al. 2009; Lindmark-Mansson et al. 2006; Sarikaya et al. 2004). Enzyme-linked immunosorbent assay (ELISA) was also applied for detecting and quantifying SCs, for example, by using the specific antibodies of PMNs. O’Sullivan et al. (1992) suggested to use a direct capture ELISA to diagnose bovine mastitis. In order to separate each type of SCs, immunomagnetic separation has been used for labeling the cell subpopulation. This method was successfully applied to isolate epithelial cells from bovine milk (Boutinaud et al. 2008), macrophages, and PMNs from sheep milk (Albenzio et al. 2009).

Recent advances of SC studies by flow cytometry in medicine diagnostic field give opportunities to study SCs in dairy field at the subpopulation level (Albenzio and Caroprese 2011; Leitner et al. 2000a; Reuter et al. 2014). The combination of immunofluorescence and flow cytometry allows the differentiation of cell types using specific antibodies (Kelly et al. 2000; Park et al. 1991; Riollet et al. 2001). Flow cytometry-cell sorting technologies distinguish and separate differential cells with more precise detection (Dosogne et al. 2003; Piepers et al. 2009). Compared with classical microscopy observation, flow cytometry allows counting SCs and identifying SC types with fewer samples and less time, thus allowing characterizing SCs and determining the roles of SCs in the milk.

### Various somatic cell count and composition

The SC composition (SC types and their percentages in total SCC) in milk varies depending on many factors: animal species, breed, stage of lactation, genetics, parity, day-to-day variation, diurnal variation, milking interval, time of sampling, sampling procedures, stress and trauma, management factors, and seasonal and storage procedures (IDF 466/2013). The macrophage and PMN percentages showed an opposite trend during different stages of lactation in ewes, the highest macrophage level observed in early lactation and the highest PMN level in late lactation (Albenzio et al. 2009). In healthy bovine and ovine milk, macrophages are generally the predominant cells, while in both healthy and unhealthy goat milks, PMNs are predominant (Dulin et al. 1983; Ostensson 1993). Nevertheless, there are several contradictory examples concerning the predominant cell type in the case of healthy bovine milk, macrophages, lymphocytes, or epithelial cells becoming the main cell type according to the authors (Table 1).

Both SC count and composition are related to milk quality, but their relationship is not necessarily associated, except in the case of high SCC corresponding to a high amount of PMNs in bovine milk. SCC gives only the total amount of cells present in milk, but not the distribution of each cell type, making it difficult to have a relevant view of cell composition. Hence, milk with various SCCs and various SC compositions has a particular fingerprint that can lead to different final characteristics of dairy products, and this will be discussed in Section 4.

## Endogenous enzymes from somatic cells

Besides protective functions in milk, SCs also provide numerous enzymes mentioned as SC endogenous enzymes in this review. These SCs as well as their endogenous enzymes have different profiles in terms of type, specificity, and activity and can be released into milk and further influence dairy processes and the quality of products. It is worthy to note that these SC endogenous enzymes are only part of all enzymes in milk, usually called milk indigenous enzymes. Actually, these indigenous enzymes in milk are well studied (Kelly et al. 2006; Kelly and Fox 2006; Kelly and McSweeney 2003), in particular the plasmin system (Ismail and Nielsen 2010). In this review, we will focus on SC endogenous enzymes.

### Somatic cells: an important source of enzymes

SCs are an important source of endogenous proteins, including enzymes. A large range of enzymes are released into milk after the lysis of SCs, and among them, lipases (e.g., lipoprotein lipase), oxidases (e.g., catalase and lactoperoxidase), glycosidases (e.g., lysozyme), and proteases (e.g., cathepsins, elastase, and collagenase). The recent advances in proteomic methods have given rise to the identification of numerous proteins from SC (Jethwaney et al. 2007; Lippolis and Reinhardt 2005). Comparison of proteomes from macrophages (Dupont et al. 2004) and milk fat globule membranes (Smolenski et al. 2006) shows that they have many proteins including enzymes in common: annexin, vimentin, and apolipoprotein, α-enolase, heat shock protein, actin, capping protein (involved in cell motility), NADP1-dependent isocitrate dehydrogenase. Even if such similarities could be partly due to the extraction of proteins in the fat layer in which SCs can be trapped and lysed at high-speed centrifugation (Smolenski et al. 2006), we cannot exclude the release of these proteins in milk as a natural phenomenon or during technological processes that can lead to underestimating their role in the final quality of dairy products.

It is only recently that a proteomic approach of the mammary epithelial cells coupled with RT-PCR and Western blotting has been developed, to give an insight into the proteins originated from this cell type in the milk and their expression (Janjanam et al. 2013). Four hundred ninety-seven proteins were identified, and some of them were identical to those originating from milk fat globule membrane (37 %) and in lactating mammary tissue (54 %), while 247 represented new mammary epithelial cell proteins.

The presence of SC endogenous enzymes in milk also suggests the occurrence of leakage or secretion of these endogenous enzymes from SCs (Kelly and Fox 2006). This is the case for cathepsin D, an endogenous enzyme from SCs detected in skim milk or whey (Larsen et al. 2006). It can degrade intracellular proteins and participate in extracellular proteolysis when it is secreted out of SCs (Briozzo et al. 1988). Dupont et al. (2004) confirmed the secretion of some SC endogenous enzymes from macrophages. The main endogenous enzymes from SC in milk are generally from macrophages and PMNs. The location of these endogenous enzymes prior to release into milk is given in Table 2.

**Table 2:** Location of main endogenous enzymes in somatic cells

Type of enzymes	Type of somatic cells				
	Macrophages	PMNs	Lymphocytes	Epithelial cells	Cell type unknown
Proteases					
Cathepsin B	+ Guha and Padh (2008)	+ Baggiolini et al. (1978); Magboul et al. (2001); Travis and Fritz (1991)		+ Lah et al. (1996); Guha and Padh (2008)	
Cathepsin C		+ Travis and Fritz (1991)			
Cathepsin D	+ Cohn (1975); Diment et al. (1988); Guha and Padh (2008)	+ Baggiolini et al. (1978); Cohn (1975); Owen and Campbell (1999)		+ Cohn (1975); Lah et al. (1996); Lkhider et al. (2004); Seol et al. (2006); Guha and Padh (2008)	
Cathepsin H	+ Guha and Padh (2008)				
Cathepsin K					+ Moatsou (2010)
Cathepsin L	+ Guha and Padh (2008)	+ Travis and Fritz (1991)		+ Lah et al. (1996)	
Cathepsin G	+ Considine et al. (2002); Campbell et al. (1989)	+ Baggiolini et al. (1978); Considine et al. (2002); Dewald et al. (1975)			
Cathepsin S	+ Guha and Padh (2008)	+ Owen and Campbell (1999)			
Elastase	+Owen and Campbell (1999); Prin-Mathieu et al. (2002); Campbell et al. (1989)	+ Baggiolini et al. (1978); Considine et al. (2000); Dubin et al. (1994); Owen and Campbell (1999); Travis and Fritz (1991); Prin-Mathieu et al. (2002)	+ Prin-Mathieu et al. (2002)		
Other enzymes					
Catalase					+ Kitchen (1976)
Lipoprotein lipase	+ Azzara and Dimick (1985b)	+ Azzara and Dimick (1985b)			
Collagenase	+ Owen and Campbell (1999); Prin-Mathieu et al. (2002)	+ Verdi and Barbano (1991); Prin-Mathieu et al. (2002)			
Acid phosphatase					+ Kitchen (1976); Andrews and Alichanidis (1975)
Myeloperoxidase	+ Owen and Campbell (1999)	+ Mukherjee et al. (2004)			

Most of the endogenous enzymes from SCs are not identified in milk and, among them, proteolytic enzymes. In fact, due to a high-dynamic range in a concentration of proteins in milk that can vary by at least a factor of 10^6^ between caseins and other minor proteins (Gagnaire et al. 2009), most of the proteolytic enzymes from SC are in a concentration that is so low that they are only detected through their activity or immunological studies (Magboul et al. 2001). This part will be discussed below. Additionally, the kinetics of SC death and the dynamics of enzyme release are difficult to measure and still unknown in milk and a fortiori in dairy products.

### Proteolysis of caseins by endogenous enzymes from somatic cells

Among the numerous endogenous enzymes from SCs, proteases are by far the most studied. They are active on caseins in milk as well as during transformation processes. They can therefore modify casein degradation of dairy products, give different textural and organoleptic characteristics to final dairy products, and even reduce the cheese yield (Grappin et al. 1999).

To determine the activity of SC proteases in milk, two strategies are often used: (i) addition of commercially available proteases to milk proteins and study of protein degradation (mainly caseins) and (ii) testing activities of commercial proteases either on caseins or synthetic substrates with or without the addition of inhibitors. Proteolytic activities of commercially available cathepsins B, D, and G and elastase as well as their enzymatic specificity (sites of cleavages) have been determined on α_s1_- and β-caseins (Considine et al. 1999, 2000, 2002, 2004; Hurley et al. 2000; Kelly and McSweeney 2003; McSweeney et al. 1995). Some other authors (Magboul et al. 2001; Somers et al. 2003) used enzymes extracted from acid whey, or they directly studied the proteolytic activities of endogenous enzymes in milk with different SCCs. Endogenous proteases from SCs, such as cathepsins B and G and elastase, were confirmed as responsible for the hydrolysis of α_s1_- and β-caseins. Consequences on casein hydrolysis were recently shown after exposure to lipopolysaccharide from the cell walls of gram-negative *E. coli*, inducing an increase in SCC without bacterial damage (Hinz et al. 2012; Olumee-Shabon et al. 2013). Thus, casein proteolysis was higher in bovine (Hinz et al. 2012) than in caprine milk (Olumee-Shabon et al. 2013), and the main casein hydrolyzed was both α_s1_- and β-caseins in bovine and caprine milk. At least, plasmin, elastase, and cathepsins B and D were shown to participate in the casein degradation.

Cathepsin D can hydrolyze all caseins (α_s1_-, α_s2_-, β- and κ-caseins) and was more active on α_s1_-caseins than on the other caseins with a broad range of cleavage sites (Hurley et al. 2000; McSweeney et al. 1995). It showed cleavage sites similar to chymosin, the main active protease from rennet used in cheese-making process, responsible for the coagulation of milk and causing the milk to separate into curds and whey. Furthermore, cathepsin B has common cleavage sites with cathepsin D and chymosin, notably on Phe_23_–Phe_24_ bond of α_s1_-casein (Magboul et al. 2001).

### Various profiles of endogenous enzymes from somatic cells

SC endogenous enzymes show different activities according to their initial location in the cell types (Table 2). The activity of cathepsin D in induced alveolar macrophages was detected to be 60-fold higher than that in PMNs (Cohn 1975), while it was not detectable in lymphocytes (Barabasi and Nassberger 1994). Due to different SC counts and compositions during lactation, the activities of cathepsin D, cysteine proteases (e.g., cathepsin B), and another unidentified milk proteinase measured in milk from the same group of cows fluctuate during lactation (Larsen et al. 2006). Additionally, the presence of some SC endogenous enzymes in milk can change the activity of other enzymes in milk. For example, plasminogen activators, of which one is associated with SC, can modify plasmin activity (Albenzio et al. 2004; Politis and Ng-Kwai-Hang 1988). Such an increase in plasmin activity was observed for SCC below 1 × 10^5^ cells.mL^−1^ and represented about 42 % of that observed with high SCC >6 × 10^5^ cells.mL^−1^ (Le Roux et al. 2003). However, as SC types present in milk were generally not determined, enzymatic activities in milk were considered as heterogeneous (Santos et al. 2003).

The profiles of endogenous enzymes in terms of type, quantity, and activity can be influenced by different SC counts and compositions in milk. Nevertheless, their relationship during milk treatments, cheese processing, and cheese quality is still unknown. The activity of SC proteases was enhanced, while SCC increased (Kelly et al. 2006; O’Farrell et al. 2002). Elastase, one protease mainly present in PMNs, has significantly higher activity when PMNs are massively recruited into milk during infection (Le Roux et al. 2003). The elastase activity was also illustrated to have a positive correlation with SCC in caprine milk (Santillo et al. 2009). Cathepsin G and lipoprotein lipase activities were detected in high-SCC milk (SCC 1.2 × 10^5^–2 × 10^6^ cells.mL^−1^), correlated with subclinical or clinical mastitis (Azzara and Dimick 1985b). Regarding lipolysis, the lipoprotein lipase is still active after pasteurization and participates in the production of free fatty acid in milk during storage. In addition, pasteurized milk with high SCC is more susceptible to lipolysis than that with low SCC (Santos et al. 2003).

Milk with various SC counts and compositions including different profiles of endogenous enzymes leads to different patterns of proteolysis and lipolysis and finally offers different characteristics of final dairy products. However, the milk in dairy industries, mixing the milk from hundreds or thousands of cows, can minimize the variations between milks in contrast to the milk mixed from several cows in the herd and a fortiori the milk from an individual cow and the quarter milk from the individual’s mammary gland. It is still difficult to ascertain a clear relationship between enzyme activities and types of SCs present in milk and to what extent one cell type would be preferable in milk to enhance final dairy product quality.

## Role of somatic cells and their endogenous enzymes in dairy processes and products

The term “effect of SCs” is mentioned in several studies (Chen et al. 2010; Fernandes et al. 2008; Ma et al. 2000; Politis and Ng-Kwai-Hang 1988; Santos et al. 2002). However, its definition is too imprecise by far. The effect of SCs in these studies includes two aspects: (i) the direct action of SCs themselves and that of their endogenous contents and (ii) the consequence of concomitant high levels of invading bacteria inducing major compositional and physicochemical changes of milk with different modified SCC levels in milk (Albenzio et al. 2011; Somers et al. 2003). As the latter aspect is usually associated with inflammation of the mammary gland, the effect of SC is generally ill-defined leading to confusion with other cofactors, recognized as negative for the researchers and dairy farmers.

In this part, the role of SCs and their impacts on dairy products will be considered. As these literature data concern the effect of SCs in a general way, it is difficult to focus only on the effective role of SCs excluding the influence of other factors associated in the experiments. Hence, these literature data will be discussed here in two parts: in Section 4.1, in which data clearly indicate the participation of some individual endogenous enzymes from SCs on dairy processes and products, and in Section 4.2, in which data concern the general effect of SCs. In addition, different methods of SC preparation used in these studies will be compared in order to bring out a new approach to study the effective role of SCs in the dairy field in the future.

### Participation of endogenous enzymes from somatic cells

The milk indigenous enzymes (including SC endogenous enzymes) were indicated as active in the udder in which the temperature is optimal for most mammalian enzymes. They continued to accumulate their enzyme activities even if these activities could be weak during the refrigerated storage (dairy farm or plant) and modified the characteristics of dairy-processed products (Kelly and Fox 2006). The roles of SC enzymes have been underestimated until now because of the presence of other numerous enzymes in milk: (i) enzymes coming from the blood and present in milk, such as plasmin; (ii) added enzymes during the cheese-making process such as calf rennet (chymosin and pepsin) or fungal rennet; and (iii) enzymes produced by microflora which play an essential role in the final characteristics of cheese, such as texture and flavor. The direct action of SC endogenous enzymes, in particular cathepsin D, in dairy processes and products will be discussed below.

#### Cathepsin D in cheese

Among the enzymes from SCs, cathepsin D is one of the most studied endogenous enzymes in milk and different types of cheeses. It is a lysosomal acidic protease, which has been greatly discussed in the human medical domain for more than a century because of its multifunctions such as a tumor marker and a cancer indicator (Benes et al. 2008; Minarowska et al. 2008). In the dairy field, studies have increased over the past two decades.

The amount of cathepsin D was measured for the first time by Larsen et al. (1996) in bovine skim milk at 0.4 μg.mL^−1^ level, mainly located in whey at 0.3 μg.mL^−1^ level. It is four- to sevenfold higher in small ruminant milk about 1.8–2.6 μg.mL^−1^ (Albenzio et al. 2009; Santillo et al. 2009). Five molecular forms were identified in bovine milk (Larsen and Petersen 1995): the two inactive forms preprocathepsin D (ppCD) and procathepsin D (pCD) and the three active forms pseudocathepsin D (pdCD), single-chained cathepsin D and two-chained cathepsin D (heavy- and light-chained cathepsin D). In bovine milk, the main form is the inactive form pCD which becomes the active form pdCD under acidic medium conditions (Larsen et al. 1993). The amino acid sequence and three-dimensional structure are close to that of two other aspartic proteinases, chymosin and pepsin (Baldwin et al. 1993). The activator-inhibitor system of cathepsin D is only partly known (Minarowska et al. 2009). Moreover, cathepsin D is able to survive most heat treatments applied during cheese manufacture (Table 3). It is also baroresistant (Moatsou et al. 2008) and stable over a range of acid pH values, from pH 3.5 to 7 (Lee et al. 1998).

**Table 3:** Variation of cathepsin D activity with different heat treatments

Heat treatment		Residual activity (%) at optimum pH	Dairy matrices	References
Temperature (°C)	Duration			
72	15 s	8	Skim bovine milk	Hayes et al. 2001
		>50	Milk serum/caseins	Larsen et al. 2000
72	60 s	~50	Milk serum	Larsen et al. 2000
		~65	Milk caseins	
65	30 min	<10	Buffer solution	Larsen et al. 2000
55	30 min	45	Skim bovine milk	Hayes et al. 2001

The presence and the activity of cathepsin D have also been illustrated in experimental rennet-free cheese and hard-cooked cheese, in which the rennet is absent or inactivated, respectively (Cooney et al. 2000; Garnot and Mollé 1987; Igoshi and Arima 1993). The presence of intact pCD and an active form derived from pCD was indicated to participate in the proteolysis process of experimental rennet-free UF-Feta cheese (Larsen et al. 2000). Furthermore, the coagulation activity of cathepsin D was confirmed in ewe milk (Albenzio et al. 2009) and in Cheddar cheese (Hurley et al. 1999). According to Santillo et al. (2009), cathepsin D concentration in caprine milk was negatively correlated with casein and protein level, possibly as a consequence of the hydrolysis capability of this enzyme. In caprine milk, cathepsin D activity was not correlated with SCC in contrast to bovine milk in which this link has been shown (Cooney et al. 2000; Kelly 1999; O’Brien et al. 2001).

#### Other endogenous enzymes from somatic cells in dairy processes and products

Due to the various endogenous enzymes from SCs (shown in Section 4.2) and the various physicochemical conditions encountered during cheese making and ripening, the influence of numerous SC enzymes on the final quality of dairy products could have been underestimated. Cathepsin B has similar cleavage sites to cathepsin D and chymosin; moreover, more than 20 % activity of cysteine protease activity remains after heat treatment at 55 °C for 30 min or 72 °C for 30 s (Magboul et al. 2001). However, to our knowledge, there are few studies concerning the state of SCs and their endogenous enzymes during the dairy process and the contribution of SC endogenous enzymes in varied ranges of dairy products. It will be interesting to elucidate on their amount and forms trapped in the cheese curds, their contribution to proteolysis in cheese varieties, and the mechanism of their release from SCs under different milk treatment conditions such as physical, chemical, and heat treatments.

The role of other SC endogenous enzymes, such as lipoprotein lipase, is not fully understood in dairy products. Lipoprotein lipases are generally considered to be causative of flavor defaults such as “rancid” in some dairy products; they could be ignored or underestimated compared to high lipolytic activities brought by *Penicillium roqueforti* in blue cheeses; they could even be responsible for the desirable flavors (e.g., “picante” flavor) in dairy products for certain consumers. The real contribution of lipase is more difficult to ascertain, due to the numerous origins of lipolytic enzymes that can be also present in cheese. Nevertheless, the desirable but weak lipolysis observed in Swiss-type cheeses is mainly due to the ripening flora *Penicillium freudenreichii* rather than to the lipoprotein lipase being rapidly inactivated during heat treatment of cheese making (Dherbécourt et al. 2010).

### Role of somatic cells

Before discussing the effective role of SCs in the dairy field, it is of great importance to understand the methodologies used in literature, such as types of milk, methods of preparing SCs, and to what extent such approaches can influence final results and interpretation. This part will primarily discuss different approaches used to study the effect of SC as shown in Table 4.

**Table 4:** Five general approaches for the study on the effect of somatic cells on dairy processes and products

Methods	Description of methods	Advantages	Inconveniences	References
(i)	Using individual milk with different SCC levels	Avoiding mixing different milk	Individual factors involved	Ma et al. (2000); Politis and Ng-Kwai-Hang (1988); Santos et al. (2002)
(ii)	Mixing healthy low-SCC milk with mastitic high-SCC milk	Easy and accurate to obtain different SCCs	Mastitic factors involved	Cooney et al. (2000); O’Farrell et al. (2002); Rogers and Mitchell (1994)
(iii)	Using originally mixed milk and classifying in different SCC categories	Classification of milk SCC is more reasonable	Individual and mastitic factors involved	Albenzio et al. (2004), (2011); Chen et al. (2010); Fernandes et al. (2008); Jaeggi et al. (2003); Somers et al. (2003)
(iv)	Isolating SCs from milk and studying their impact in the dairy products	Avoiding other causative factors in the study of SC roles	Limited quantity of SCs	Albenzio et al. (2009); Azzara and Dimick (1985a, b); Caroprese et al. (2007)
(v)	Concentrating SCs from milk and then adding these SCs with different quantities into milk with low SCC or without SCs	Avoiding other causative factors in the study of SC roles	Limited quantity of SCs	Marino et al. (2005); Sanchez-Macias et al. (2013)

#### Different approaches to prepare somatic cells and to study their effect

(i) Use of individual milk or quarter milk
  This method is based on separately collecting individual milk or quarter milk. There are few studies carried out with this method due to great differences between individual milk samples in terms of milk composition and SC content. In the case of high SCC (>10^6^ cells.mL^−1^), the effect of SCs takes into account other cofactors such as differences between milk samples and the contaminants in milk leading to mastitis. The effect of SCC and the stage of lactation on the quality of various products were studied, such as raw milk, ultrahigh-temperature milk, Cheddar cheese, and full cream milk powder (Auldist et al. 1996a, b, c). One study involving injection of *Streptococcus agalactiae* in a quarter of milk compared the shelf-life of milk containing low and high SCC on the same cows before and after infection (Ma et al. 2000). The low-SCC milk kept a high organoleptic quality during cold storage, even 21 days of shelf-life after pasteurization, in contrast to the high-SCC milk that became rancid, bitter, and astringent. This was correlated with higher lipolysis and proteolysis in high-SCC milk.
(ii) Mix of milk from healthy and mastitic cows
  Mixing healthy cow milk containing low SCC with mastitic cow milk containing high SCC in different proportions allows the different SCC levels to be obtained easily and accurately (Cooney et al. 2000; O’Farrell et al. 2002; Rogers and Mitchell 1994). It is difficult to extract information regarding the effective role of the SC from these experiments, since the resulting milk combined the composition of healthy and mastitic milk as well as the various SC counts and compositions. Rogers and Mitchell (1994) found that the organoleptic grade of yoghurt made with low-SCC milk (<2.5 × 10^5^ cells.mL^−1^) was superior to that manufactured from high-SCC milk (>5 × 10^5^ cells/mL-1). The quality of Swiss-type cheese, e.g., cheese moisture, salt, pH, and eye production during ripening, was unaffected by SCC, and the patterns of proteolysis in cheese were altered, but this may be due to a number of factors rather than SCC (Cooney et al. 2000).
(iii) Use of originally mixed milk
  Collecting the originally mixed milk with different SCCs and classifying it into different SCC categories has often been used in recent years. Nevertheless, it involves both individual differences of milk and other causative factors in the results of the effect of SCs. To have information on the effective role of SCs, interpretation has to be focused on the results of healthy low-SCC experiments. It was shown that Prato cheese made with low-SCC cow milk (<2 × 10^5^ cells.mL^−1^) had a lower number of yeasts and molds, which usually cause deterioration of products (Vianna et al. 2008). Albenzio et al. (2004) have shown that the cheese curds made from low-SCC ewe milk (<5 × 10^5^ cells.mL^−1^) contribute to improve the sensory properties of Canestrato Pugliese cheese during ripening. Additionally, SC count and composition were both parameters associated with the enzyme evolution of milk that contributes to a proteolytic pattern of ewe milk and to cheese sensory quality. For example, macrophages, by producing urokinase-plasminogen activators, were able to regulate the plasminogen-plasmin system that strongly influences cheese ripening.
(iv) Isolating somatic cells from milk
  Isolating SCs from milk and then adding them to healthy milk seem perfect to study the effective role of SCs on milk and dairy products as well as selecting one or more cell types and their endogenous enzymes according to the origin of the cells used. The simple method of centrifugation and the advanced methods such as cell sorting flow cytometry and magnetic separation were applied for the cell isolation. Nevertheless, a relatively elevated quantity of milk is required in the case of low-SCC milk from healthy animals to obtain an adequate quantity of SCs. This may be the reason why the study on the effect of SCs until now has been only carried out on milk proteins, rather than on dairy products.
  By centrifugation, SCs were isolated as a cell pellet from milk and were prepared in a cell suspension for cell analysis. Nevertheless, only one fourth or less of all SCs in milk was obtained in the precipitated pellet (Prescott and Breed 1910), and whether the small fraction in cell pellet has a fairly constant proportion of whole cells is still a question. By using Ficoll discontinuous density gradient, SCs were isolated from the milk of healthy or mastitic cows. Macrophages were then separately maintained in cell cultures to secrete lipolytic enzymes into the medium. These secreted lipolytic enzymes bind to the fat globule membranes in milk and expose the fat to degradation over storage. The other cell types, PMNs and lymphocytes, did not show lipoprotein lipase activity (Azzara and Dimick 1985a, b). Recently, using a magnetic positive separation method, Caroprese et al. (2007) have isolated macrophages from sheep milk and shown their proteolytic ability on α_s_-caseins (~20 %), β-casein (~10 %), and minor degradation on γ-casein (~1 %). Using the same method, Albenzio et al. (2009) isolated not only macrophages but also PMNs from sheep milk. It has been shown that PMNs release lysosomal enzymes that induce a more intense hydrolysis on casein in late rather than in early lactation and positive correlation between these endogenous enzymes (e.g., cathepsin D) and clotting time.
(v) Concentrating somatic cells in milk
  Concentrating SCs from milk and then adding different quantities to milk with low SCC or without SCs seem like a more feasible method to study the effective role of SC on milk and on cheese as well. This is a way to collect SCs with a peculiar composition including mainly macrophages, lymphocytes, or PMNs rather than to collect only PMNs from mastitic milk and also to vary the SCC with the same cell type. To our knowledge, Marino et al. (2005) were the first to study the contribution of SC and their proteolytic enzymes in Cheddar-type cheese by adding different quantities of concentrated SCs in a milk containing low SCs (<150,000 cells.mL^−1^) for cheese making. The milk used to concentrate SCs was collected from milk with >10^6^ cells.mL^−1^ to obtain a necessary quantity of SCs. Their results suggested that SCs have a direct influence on cheese moisture content and contribute to proteolysis in milk and Cheddar-type cheese. As mentioned in Section 3.3, the milk from mastitic and healthy cows with different SC counts and compositions has different profiles of SC endogenous enzymes. The cow milk used in this experiment was mastitic so it was mainly composed of PMNs; as a result, the effect of SCs is supposed to be interpreted as the “effect of PMNs.” Recently, the effect of SCs concentrated from healthy goat milk was studied on low-fat cheese in order to improve cheese sensorial quality (Sanchez-Macias et al. 2013). Only miniature fresh goat milk cheeses were made in these experiments due to the limited quantity of collected SCs. Even if the ripening period lasted only 7 days, a general reduction in casein fractions was observed in the cheese with added SCs: β-casein (43 vs 26 %), para-κ-casein (37 vs 23 %), α_s2_-casein (30 vs 20 %), and α_s1_-casein (25 vs 14 %). Different patterns of casein degradation were found, while SCC was increased in raw or pasteurized goat milk cheeses. Moreover, cheeses with added SC were found to have softer texture and incremental color without any modification of chemical composition (Sanchez-Macias et al. 2013). Even if the SC composition was not determined in this study, the main SCs in healthy goat milk are PMNs (Dulin et al. 1983; Ostensson 1993), which are the same predominant cell type in mastitic cow milk (Azzara and Dimick 1985b). Until now, no study on the effective role of SCs on milk and on cheese has been conducted with healthy cow milk.

#### A new approach to study the effective role of somatic cells

It is worth noting that different milks and different methods of preparing SCs from milk can both induce different consequences on SCs themselves and then different roles of SCs on dairy processes and products. Adding isolated or concentrated SC with different ratios to cheese milk with low SCC or ideally without SCs will be a good strategy to study the effective role of SCs on dairy products. With the development of membrane filtration techniques in the dairy field (Saboya and Maubois 2000), the selective separation of SCs from raw milk becomes possible and feasible in practical experiments. Comparing other SC preparation technologies to study the role of SCs, the main advantages of membrane filtration techniques are listed below: (i) a great quantity of SCs can be efficiently concentrated at an industrial scale. Thus, collecting sufficient SCs from healthy milk originally poor in SCs is no longer a limiting step, and making diverse dairy products and analyzing the lipolysis and proteolysis in a long term become achievable; (ii) by membrane filtration, the milk deprived in SCs can be obtained by keeping the maximum native characteristics of milk. This SC-free milk can be used as the control in which SCs can be added in different quantities for cheese making. By using the same standardized milk in cheese making, the individual factors between different milk samples in other studies can be excluded; (iii) SCs can be added in different quantities to SC-free milk. Even with a high-added SCC level, the mastitic factors in other studies can be also excluded in this new approach; (iv) the studies on the effect of different SC types can be considered by concentrating SCs from different milk samples, for example, healthy bovine milk enriched in macrophages and mastitic bovine milk enriched in PMNs. However, several aspects of this new approach should be considered: the choice of membrane for optimized filtration, microfiltration, or ultrafiltration; the coseparation of other components in milk such as fat, proteins, and bacteria; and the influence of membrane filtration on the SCs, and to what extent, the final dairy products are affected.

## Conclusion and perspectives

Although different approaches to study the effect of SCs have been conducted, few protocols have been undertaken to highlight the effective role of SCs on dairy products without the other concomitant factors in high-SCC milk. In this review, a new approach to study the effective role of SCs on dairy processes and products is suggested by comparing relevant methods of preparing SC. Isolating or concentrating SCs from milk and adding these SCs at different ratios in SC-free milk seems to be a good strategy. The milk status (mastitis or not mastitis and subclinical, chronic, or acute mastitis), SCC, and SC profiles should be precisely defined. This would allow the control of SC count and composition and, therefore, their endogenous enzyme profiles in milk. Such a fingerprint would help to characterize more precisely the SC impact on technological properties and final quality of dairy products besides the initial microbiological and nutritional characteristics of milk.

By analyzing the literature data, we have pointed out in this review that SCs can have a desirable role, in terms of acceleration of proteolysis in the cheese-ripening process and improvement of cheese sensory quality. Many studies remain to be done to fully understand the effective role of SCs, each cell type, and their extent in the dairy field. In particular, the relationship between SCs and their endogenous enzymes as well as the mechanism by which endogenous enzymes are released from SCs under different conditions and during dairy processes need to be elucidated.
